# Dataset of the survey on e-registration and geo-referenced of rice value chain actors for the diffusion of technologies: Case of Benin and Côte d'Ivoire

**DOI:** 10.1016/j.dib.2020.105642

**Published:** 2020-04-30

**Authors:** Aminou Arouna, Rachidi Aboudou

**Affiliations:** Africa Rice Center (AfricaRice), 01 BP 2551, Bouaké 01, Côte d'Ivoire

**Keywords:** Rice value chain actors, census, development of agricultural technologies, improved rice varieties, out-scaling, production systems, West Africa

## Abstract

The paper presents a dataset of the e-registration of rice value chain actors in Benin and Côte d'Ivoire for assessing the adoption of innovations and the diffusion of new rice technologies. Data were collected from actors after a census conducted in three steps. In the first step, main rice production regions and rice value chain actors were identified. In the second step, we updated the list of actors based on membership of actors’ associations. In third step, we did the census of all individual actors and geo-localized all farmers’ fields and villages using GPS device. Data were collected for the 2018 growing seasons. The dataset contains 17,639 observations (9,000 in Benin and 8,639 in Côte d'Ivoire) with 159 variables divided into six sections: (i) preliminary information on the respondents; (ii) socio-economic characteristics; (iii) information on the rice plots; (iv) knowledge, use and access to rice varieties; (v) knowledge, use and access to agricultural equipment and methods; and (vi) information on post-harvest activities. Six categories of actors were identified: foundation seed producers (420), certified seed producers (1,212), paddy rice producers (14,230), parboilers (1,735), millers (188) and traders (1,429). The dataset is available online at Mendeley data repository. The dataset is valuable for the diffusion at large scale of improved technologies and an effective monitoring of the dissemination. Data can be used by scientists to have better understanding of the rice value chains, rice production systems, the level of knowledge, accessibility and adoption of improved rice varieties and agricultural technologies, for further research regarding rice value chain development, technologies testing and socioeconomics study of rice value chain actors. Because of the large number of observations (17,639), data can be used as sampling frame for further experiment or surveys based on random samples. Moreover, the dataset has the potential of generating descriptive statistics at the most disaggregated level of administrative units or villages for different equipment, methods and varieties adopted by gender and country.

**Specifications table**

**Table:** 

Subject	Social Sciences
Specific subject area	Agriculture, varieties adoption, agricultural equipment and methods use, yield, ecology
Type of data	Table Figure Data in Excel format & STATA format (.dta)
How the data were acquired	Data were collected through census and surveys of rice value chain actors with structured questionnaire using android tablet.
Data format	Raw Analyzed Cleaned
Parameters for data collection	Face-to-face interviews using structured questionnaire and geographic locations obtained with GPS device.
Description of data collection	Census of all rice value chain actors were done in three steps. In the first step, main rice production regions and rice value chain actors were identified. In the second step, we updated the list of actors based on memberships of actors’ associations. Finally, we did the census and interviewed all actors and geo-localized farmers’ fields and villages using GPS device.
Data source location	The data were collected in southern and northern parts in two countries: 1. Benin 1.1. Southern part of Benin (including 14 districts: Bante, Savalou, Dassa Zoume, Ouesse, Save, Glazoue, Zogbodomey, Houeyogbe, Dangbo, Zagnanando, Ouinhi, Grand Popo, Cove and Adjohoun) 1.2. Northern part of Benin including Malanville district 2. Côte d'Ivoire 2.1. Southern part of Côte d'Ivoire (including Goh-Djiboua and Gagnoa regions) 2.2. Northern part of Côte d'Ivoire (including Gbeke and Hambol regions)
Data accessibility	Repository name: Mendeley Data Data identification number: N/A Direct URL to data: https://data.mendeley.com/datasets/53bg88r72f/draft?a=d1748bfe-10e0-4c0e-9b5d-8649d906881d

**Value of the data**

• The data in this article is useful because it is a large multidisciplinary dataset comprising 17,639 observations of six different categories of actors (foundation seed producers, certified seed producers, paddy rice producers, parboilers, millers and traders) for better understanding of the rice value chains, rice production systems and adoption of improved rice varieties and agricultural technologies.
• This dataset can be used by scientists, policy makers, extensions officers, NGO and development agencies such as United Nations' organizations.
• The data is valuable for further research regarding rice value chain development, socioeconomics study of rice value chain actors, yield analysis (spatial distribution and yield gap), knowledge, accessibility and adoption of rice improved varieties and technologies [1] and to analyse rice cropping systems [2] . The dataset can be used to map and characterize rice value chain actors in West Africa and to develop appropriate technologies along the rice value chains. The dataset can be further analyzed using advanced methods (e.g. econometric models, spatial analysis).
• The data is valuable for diffusion at large scale of improved technologies and an effective monitoring of the dissemination.
• Because of the large number of observations (17,639), dataset is valuable as sampling frame for future experiment or surveys based on random samples.

## 1 Data description

**Fig. 1:**
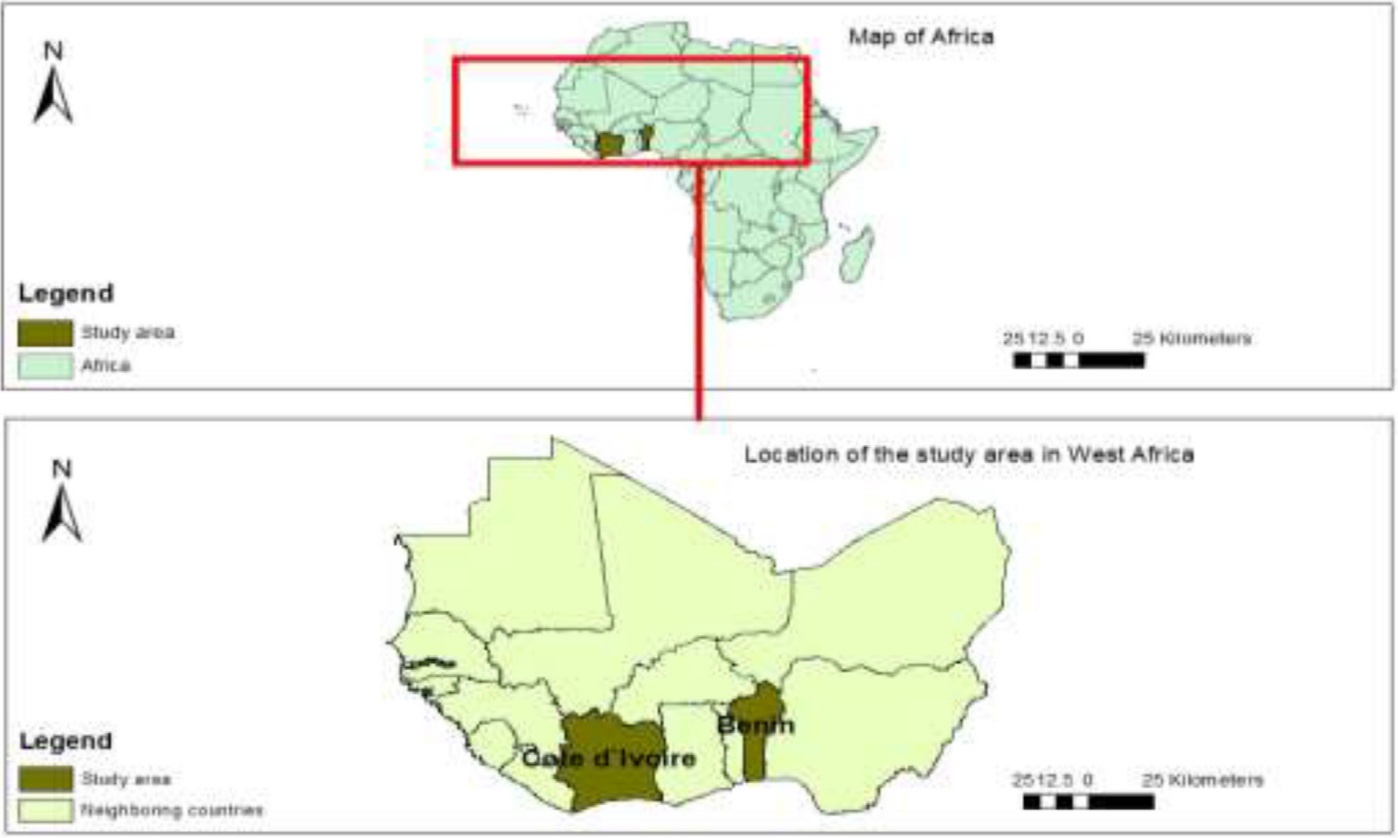
Map of the two countries

The diffusion of agricultural technologies faces enormous challenges such as the identification and the geolocation of the real actors in need [1] . Sampling frames required for surveys are often missing. In order to fill these gaps and better fit the preference of actors, a census and interviews of all rice value chain actors were conducted in two main rice production zones in two West African countries (Fig. 1).

The questionnaire which is provided on the Mendeley data repository (see the link in the specification table and in Appendix) was used to collect information on rice actors’ demographic characteristics and specific sections related to each category of actors. For producers (foundation seed producers, certified seed producers and paddy rice producers), 150 variables were grouped in five sections: preliminary information on the respondents; socio-economic characteristics; information on the rice plots; knowledge, use, and access to rice varieties; and knowledge, use and access to agricultural equipment and methods. Parboilers, millers and traders were interviewed, in addition to preliminary information on the respondents and socio-economic characteristics (sections 1 and 2), on nine questions related to post-harvest activities (section 6 of the questionnaire). Table 1 summarizes the dataset and variables. The dataset is in Microsoft Excel (in one sheet) and STATA format (Accessible on Mendeley data repository through the link in the Table of specification). The questionnaire, the Excel sheet and STATA format provide labels and variable names definition.

**Table 1:** Summary of the variables included in the dataset grouped by section

Variables	Scale type	Scale class	Source of data
**Section 1: Preliminary information on respondents**			
Code of the respondent	Numeric	Unique code	surveys
Name of country	Nominal	Benin, Côte d'Ivoire	surveys
Name of region or district	Nominal		surveys
Name of town or village	Nominal		surveys
Date of survey	Numeric		surveys
**Section 2: Socio-economic characteristics of respondents**			
Name and surname of the actor	Nominal		surveys
Age	Numeric		surveys
Gender	Nominal	Female, Male	surveys
Education level attended	Ordinal	Illiterate, Primary, Junior high school, Senior high school, University.	surveys
Number of household members producing rice (Having a rice field)	Numeric		surveys
GPS coordinats	Numeric		surveys
Telephone number of the respondent	Numeric		surveys
Type of actors	Nominal	Foundation seed producers, certified seed producers, Producers of rice for consumption (paddy rice producers), Parboilers, Millers, Traders.	surveys
**Section 3: Information on the rice plots**			
Name of the plot	Nominal		surveys
Type of ecology	Nominal	Rainfed upland, Lowland rainfed, Irrigated upland, Mangrove	surveys
Cultivated variety for the first season	Nominal	NERICA, IR841, ARICA, SAHEL, WITA, FARO, BL, NL, BOUAKE, JT11, CHINOIS.	surveys
Rice area for the first season	Numeric		surveys
Production for the first season	Numeric		surveys
Cultivated variety for the second season	Nominal	NERICA, IR841, ARICA, SAHEL, WITA, FARO, BL, NL, BOUAKE, JT11, CHINOIS.	surveys
Rice area for the second season	Numeric		surveys
Production for the second season	Numeric		surveys
**Section 4: Knowledge, use, access to rice varieties**			
Name of variety	Nominal	NERICA, IR841, ARICA, SAHEL, WITA, FARO, BL, NL, BOUAKE, JT11.	surveys
Knowledge of the variety	Nominal	Yes, No	surveys
Name of the variety with its code if applicable	Nominal		surveys
Access to variety	Nominal	Yes, No	surveys
Grown at least once	Nominal	Yes, No	surveys
Grown the variety in 2018	Nominal	Yes, No	surveys
**Section 5: Knowledge, use and access to equipment and methods**			
Equipment or method	Nominal	ASI thresher (for threshing and winnowing paddy rice), GEM (for rice parboiling), RiceAdvice, Smart-valley, SRI (Intensive Rice Farming System), Manual weeder, Power tiller	surveys
Knowledge of the equipment	Nominal	Yes, No	surveys
Access to the equipment	Nominal	Yes, No	surveys
Use at least one of the equipment	Nominal	Yes, No	surveys
Use in 2018	Nominal	Yes, No	surveys
**Section 6: Information on post-harvest activities (for parboilers, millers and traders)**			
Quantity of parboiled rice per month	Numeric		surveys
Number of months of work in the year	Numeric		surveys
Knowledge of GEM equipment	Nominal	Yes, No	surveys
Access to GEM equipment	Nominal	Yes, No	surveys
Use of GEM equipment	Nominal	Yes, No	surveys
Quantity of rice milled per month	Numeric		surveys
Quantity of rice sold in the year	Numeric		surveys
Proportion of imported rice sold	Numeric		surveys
Proportion of local rice sold	Numeric		surveys

**Fig. 2:**
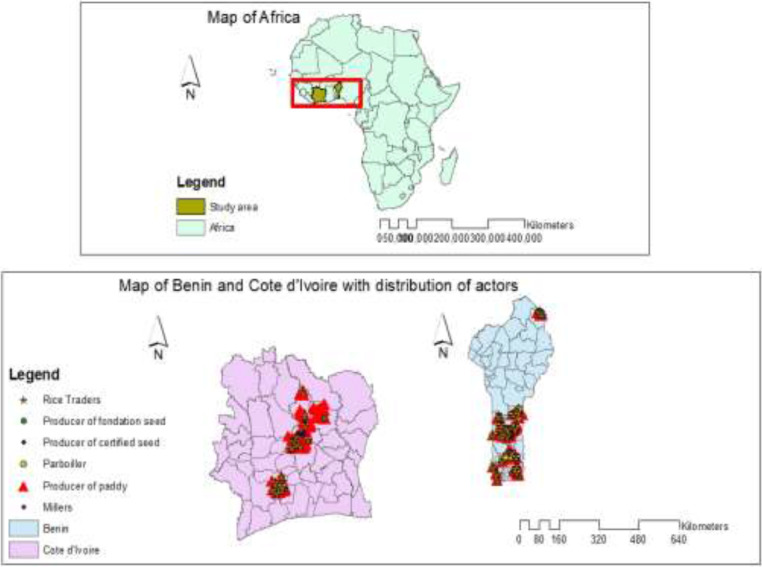
Study areas and the distribution of rice value chain actors

The data resulted from surveys of six main categories of rice value chain actors: foundation seed producers, certified seed producers, paddy rice producers, parboilers, millers and traders. A total of 17,639 rice value chain actors were interviewed and geo-localized. Data were collected for the 2018 growing seasons (first and second seasons). As an example of the potential use of the dataset, Fig. 2 shows a map representing the spatial distribution of the different rice actors in Benin and Côte d'Ivoire. The shape file of the Fig. 2 is accessible on Mendeley data repository through the link in the Table of specification.

Table 2 shows the distribution of the respondents by category, gender and country. In the population of actors in the two countries, 90.62% (11,503) of male and 55.15% (2,727) of female were paddy rice producers while 2.29% (291) of male and 2.61% (129) of female were foundation seed producers. Certified seed production involved about 8.16% (1,036) of male and 3.56% (176) of female. Rice parboiling activity occupied 0.95 % (121) of male and 32.64% (1,614) of female. 4.90% (622) of male and 16.32% (807) of female were traders (Table 2).

**Table 2:** Frequency of actors surveyed in Benin and Côte d'Ivoire by gender

Type of actors	Benin (N=9,000)		Côte d'Ivoire (N=8,639)		Total (N= 17,639)	
	Male (N=4,993) %	Female (N=4,007) %	Male (N=7,701) %	Female (N=938) %	Male (N=12,694) %	Female (N=4,945) %
Foundation seed producers	5.79 (289)	3.22 (129)	0.03 (2)	0	2.29 (291)	2.61 (129)
Certified seed producers	5.17 (258)	2.32 (93)	10.10 (778)	8.85 (83)	8.16 (1,036)	3.56 (176)
Paddy rice producers	83.80 (4,184)	50.99 (2,043)	95.04 (7,319)	72.92 (684)	90.62 (11,503)	55.15 (2,727)
Parboilers	2.42 (121)	39.46 (1,581)	0	3.52 (33)	0.95 (121)	32.64 (1,614)
Millers	2.44 (122)	0.12 (5)	0.77 (59)	0.21 (2)	1.43 (181)	0.14 (7)
Traders	3.93 (196)	14.40 (577)	5.53 (426)	24.52 (230)	4.90 (622)	16.32 (807)

() Frequency

Table 3 shows another use of the dataset by presenting the socioeconomic characteristics of rice value chain actors. The average age was greater than 39 years old for all actors. Millers had 46% of illiterate and the foundation seed producers had 88% of illiterate (Table 3). IR varieties were adopted in Benin by 89.41% of producers (Table 4). WITA and BOUAKE seeds were used in Côte d'Ivoire by 33.29% and 11.79%, respectively.

**Table 3:** Selected socio-economic characteristics of actors

Socioeconomic characteristics		Foundation seed producers (N=420)	Certified seed producers (N=1,212)	Paddy rice producers (N=14,230)	Parboilers (N=1,735)	Millers (N=1,88)	Traders (N=1,429)
Age		39.43 (11.39)	40.73 (10.94)	41.41 (10.99)	40.62 (8.78)	40.17 (9.84)	39.52 (9.61)
Education level (%)	Illiterate	88.10	59.90	65.02	72.10	46.28	57.03
	Primary	7.38	26.82	26.26	22.88	30.85	31.21
	Junior high school	4.05	10.48	6.80	4.67	12.23	9.66
	Senior high school	0.48	2.56	1.57	0.35	7.45	1.61
	University	0	0.25	0.34	0	3.19	0.49

() standard deviation

**Table 4:** Population adoption rates of technologies among producers

	Benin (N=6,830)		Côte d'Ivoire (N=8,032)		Total (N=14,862)	
	%	N	%	N	%	N
**Rice variety names**						
NERICA	9.37	640	1.95	157	5.36	797
IR	89.41	6107	0.00	0	41.09	6107
ARICA	0.10	7	0.06	5	0.08	12
WITA	0.00	0	33.29	2674	17.99	2674
JT	0.00	0	7.48	601	4.04	601
BOUAKE	0.00	0	11.79	947	6.37	947
**Equipment and methods**						
ASI	5.55	379	19.15	1538	12.90	1917
RiceAdvice	0.10	7	0.09	7	0.09	14
Developed inland valleys (Smart-Valley)	45.92	3136	41.71	3350	43.64	6486
System of Rice Intensification (SRI)	21.87	1494	16.53	1328	18.99	2822
Mechanical weeder	8.40	574	3.42	275	5.71	849
Power tiller	4.55	311	13.10	1052	9.17	1363

## 2 Experimental design, materials, and methods

Data were collected from rice value chain actors after a census conducted in three steps. In the first step, rice value chain actors and the regions of rice production were identified in each country. The regions selected are the major rice growing areas named hubs. These hubs are zones of high potential impact where rice research innovations are integrated across the value chains to achieve development outcomes and impact [3]. In the hubs, Africa Rice Center (AfricaRice) and national scientists are introducing, evaluating and validating new rice technologies, and work with development partners to facilitate the training of farmers, out-scaling of technologies and establishment of linkages among actors along the rice value chains. Hubs were selected in participatory approach with national partners and value chain actors. Data were collected in the two hubs selected in Côte d'Ivoire and Benin. In the second step, the list of actors was obtained from actors’ associations and updated through census. The census was conducted with the help of extension agents, from national agricultural research institutes and ministry of agricultures, who visited each household in the study area. In each area, the extension agents went from house to house to check the name of actors on the lists. If a name is missing and a household member is involved in a rice activity, then the list is updated. All individual actors were interviewed. The questionnaire and an android-based application were developed for e-registration of rice value chain actors. Finally, enumerators were recruited and trained for data collection. The questionnaire was pre-tested by enumerators before the beginning of the surveys. Data collection was coordinated and supervised by AfricaRice staffs, national agricultural research institutes in Benin and Côte d'Ivoire, and government extension officers. Actors’ leaders facilitated contact with respondents. Face-to-face interviews were conducted, and the location of all rice farmers’ fields and villages were geo-referenced using GPS device. Data were analyzed using STATA 15. The STATA syntax used for generating the tables in this paper is provided in Mendeley data repository (see link in Appendix). The dataset is available online at Mendeley data repository and it is not related to any primary research article.

Appendix. Supplementary data

Supplementary data and files to this article can be found online at https://data.mendeley.com/datasets/53bg88r72f/draft?a=d1748bfe-10e0-4c0e-9b5d-8649d906881d

## Declaration of Competing Interest

The authors declare that they have no known competing financial interests or personal relationships that could have appeared to influence the work reported in this paper.
